# The acceptability of TV-based game platforms as an instrument to support the cognitive evaluation of senior adults at home

**DOI:** 10.7717/peerj.2845

**Published:** 2017-01-03

**Authors:** Carlos Rivas Costa, Manuel José Fernández Iglesias, Luis Eulogio Anido Rifón, Miguel Gómez Carballa, Sonia Valladares Rodríguez

**Affiliations:** Ingeniería Telemática, University of Vigo, Vigo, Spain

**Keywords:** Serious games, Senior adults, Cognitive evaluation, In-home care, Smart TV, Acceptability studies

## Abstract

**Introduction:**

The computing capabilities of state-of-the-art television sets and media centres may facilitate the introduction of computer-assisted evaluation at home. This approach would help to overcome the drawbacks of traditional pen-and-paper evaluations administered in clinical facilities, as they could be performed in a more comfortable environment, the subject’s home, and they would be more flexible for designing complex environments for the evaluation of neuropsychological constructs that are difficult to assess through traditional testing. The objective of this work was to obtain some initial evidence about the technical acceptance by senior adults of serious games played at home on the TV set and therefore about the convenience of further investigating such an approach to cognitive assesment.

**Materials and Methods:**

We developed a collection of games to be deployed on a TV environment. These games were tried by a group of senior adults at their homes. The Technology Acceptance Model (TAM) was used to validate this approach. Surveys were performed to study the perceived usefulness and perceived ease of use of such technical setting as an instrument for their cognitive evaluation; that is, its technical acceptance. Subjective information collected from participants was correlated with actual interaction data captured. An additional survey was performed 36 months after pilot testing to have an indication about the long-term perceptions about usefulness and ease of use.

**Results:**

More than 90% of participating subjects perceived cognitive games on TV as useful or very useful. The majority of participants selected the TV set as their preferred option to interact with serious games at home, when compared to other devices such as smartphones, tablets or PCs. This result correlates with the number of participants perceiving them as easily usable or very easy to use, and also with automatically captured interaction data. Three out of four seniors expressed their interest in keeping the system at home after the pilot. Besides, these perceptions are fairly stable in time as shown by the survey performed 36 months after pilot testing.

**Limitations:**

Although participating users are a representative sample of the Galician population, which in turn is comparable to the population of most rural areas in Europe, a larger and more diverse user sample would be needed to obtain significant results for a wider population profile.

**Conclusion:**

The study confirmed the technical acceptance, that is, the perceived usefulness and perceived ease of use, of the TV-based home technical setting introduced as a means of cognitive evaluation. This study provides initial evidence on the viability of a TV-based serious games approach for cognitive longitudinal screening at home with little intervention of clinical professionals, thus contributing to the early detection of cognitive impairments in the senior population.

## Introduction

In the last few years we have witnessed a revolution in the development and use of information technologies, facilitating their introduction in more and more application fields and reaching more and more people (Charness & Boot, 2009). Older people was not left out of this rapid expansion, since increasingly accessible and usable technologies are being developed, even by people with very limited technological knowledge. A good example of this trend are applications developed to be accessed through interactive television (iTV). The aim of iTV applications and services is diverse, including leisure applications such as games (Rice & Alm, 2008); information services (e.g., weather forecast, news); educational tools (e.g., video clips, self-help guides); health tools (e.g., cognitive training Shatil et al., 2014), control of vital parameters (Maglaveras et al., 2003; Blackburn, Brownsell & Hawley, 2011), among others. The research discussed in this paper is aimed to study the viability of using iTV-based applications for the cognitive assessment of senior adults at home.

Cognitive assessment consists on the study of a subject’s performance in a given neuropsychological domain to detect dysfunctions or impairments (Lezak et al., 2004). Evaluation techniques and protocols have been defined and implemented for domains such as visuospatial abilities, motor coordination, language use, attention and concentration, executive functions or memory.

Typically, cognitive evaluations take place in a controlled environment, usually in a clinical facility, and they are conducted by health professionals. The tools used consist of a collection of validated neuropsychological pen-and-paper tests (Howieson & Lezak, 2010; Lezak et al., 2004). The process is based on a face-to-face interview of variable duration, depending on the characteristics of the test suite, along with a guided data collection process, and these instruments produce results in the form of a mark in scale providing an indication of the state of a person in relation to the target neuropsychological domain.

However, these tests have limitations that may compromise the reliability of the results obtained. Most relevant limitations of classic neuropsychological tests are related to the fact that senior users perceive these kind of tools as intrusive and alien (Chaytor & Schmitter-Edgecombe, 2003), which in turn affect aspects that may dramatically influence the results, like motivation, attention, alertness, or stress. Classical tools also offer a late diagnosis (Holtzman, Morris & Goate, 2011); lack ecological validity (i.e., the lack of correlation of test items with actual activities of daily living) (Knight et al., 2009; Farias et al., 2003); and results depend on confounding factors such as age, educational level (Cordell et al., 2013), or practice effect (Lezak et al., 2004; Hawkins, Dean & Pearlson, 2004).

Several research initiatives can be found in the literature aimed to develop alternate solutions to neuropsychological assessment based on the use of information and communication technologies (ICT). The incorporation of ICT allows to evaluate some signs that may precede cognitive impairments, which also can serve as early indicators, such as late onset depression or mood assessment; gait (since there is an association between reduced gait velocity, cadence, and stride length and declines in overall cognition, memory and executive functions (Mielke et al., 2013)); motor skills such as balance and weak hand grip, which is associated with an increased risk of dementia among persons with possible mild cognitive impairment (Larson et al., 2006); olfactory impairments (Stamps & Bartoshuk, 2013; Wilson et al., 2009) or speech analysis (López-de-Ipiña et al., 2013), among others. In general, we can observe two broad main approaches, namely digitalization of classical tests (Robinson & Brewer, 2016) and gamification (Tong & Chignell, 2014).

A promising ICT-based approach for cognitive assessment is the use of serious games. Note that games may not have enjoyment, entertainment or fun as their primary purpose (Michael & Chen, 2005), and therefore games were introduced in many application areas beyond entertainment such as education (Connolly et al., 2012), rehabilitation (Holden, 2005) or military training (Smith, 2009). Lately, a promising application area is cognitive evaluation, as digital games may have some advantages consequence of their computerized nature (Parsons, 2014). Testing protocols can be easily standardized, an increased accuracy in timing and response latencies can be achieved, data collection and administration is simplified, and a better randomization of the presentation of stimuli in repeated administrations is possible. Besides, these kind of games also support a precise representation of dynamic perceptual stimuli (visual, auditory, olfactory, ambulatory, and haptic) (Armstrong et al., 2013). Finally, digital games, due to their ludic nature, are an excellent alternative to traditional pen-and-paper tests for the frequent assessment of individuals at risk (Hagler, Jimison & Pavel, 2014), in other words, for a screening purpose.

Specifically, proposals have been developed to use games to assess visuospatial ability (van der Ham et al., 2015; Serino et al., 2015), episodic memory (Sauzéon et al., 2016a; Sauzéon et al., 2016b; Arvind Pala et al., 2014; Sauzéon et al., 2012), prospective memory (Banville et al., 2010), attention (Delgado et al., 2016), executive functions (Aalbers et al., 2013; Renison et al., 2012) among other constructs (Manera et al., 2015; Siraly et al., 2015). Regarding the technological aspect (i.e., the type of device and user interfaces to support video game interaction) we can observe that the vast majority of the studies referenced are based on the use of a personal computer (PC). In most cases users access to a Web application, although some desktop applications are also proposed (Sauzéon et al., 2016a; Sauzéon et al., 2016b; Sauzéon et al., 2012; Koenig & Krch, 2012). Other studies incorporate mobile devices such as smartphones (Thompson et al., 2012; Tong & Chignell, 2014) and tablets (van der Ham et al., 2015; Serino et al., 2015; Delgado et al., 2016; Pazzi et al., 2014), which facilitate mobility and access to video games regardless of the location of participants. Besides, these works testing through games was administered in a controlled environment, typically in a clinical facility or a senior day-care centre.

However, the introduction of these new game-based tests may introduce some limitations, such as the technological gap (Van Dijk & Hacker, 2003). In addition, since their validation is focused on their applicability to cognitive assessment, issues such as their acceptance level, usability or accessibility are not addressed. Given the technological divide that still exists among the senior adult population, these are not minor issues. The impact of a neuropsychological assessment model will be dramatically limited if target users have difficulties to use it, or if they just reject it.

In this study we propose as our research hypothesis that the best approach to introduce serious games for the elderly at home is the use of the TV set as the main interacting device. It is worth noting that at the time of writing this paper, contributions proposing the ubiquitous TV set as interfacing device are scarce (Shatil et al., 2014; Arvind Pala et al., 2014; Sauzéon et al., 2012), no matter that it can be considered the most familiar technological device available at home in the case of senior adults (Vázquez et al., 2012; Ofli et al., 2016; Heart & Kalderon, 2013).

In order to validate this hypothesis, two objectives were identified. On the one hand, we aimed to study the acceptability of a TV-based game platform that offers senior users the possibility of interacting with a series of digital games at home. The analysis of the data obtained from this interaction would eventually serve to carry out the cognitive assessment of the participants. Note that this latter objective, namely cognitive assessment, is not among the aims of this research. However, this proposal aims to eventually overcome the limitations of classical testing by making use of a more user-friendly tool (i.e., different to paper and pencil tests), through a popular device among senior users (i.e., the TV set) in a comfortable and relaxed environment (i.e., their homes).

On the other hand, this research was targeted to explore the possibility of using such systems as a screening mechanism that would eventually support an ecological, non-intrusive and affordable monitoring structure of the cognitive status for our target population group. This aspect aims to advance in the early identification of cognitive problems to contribute to overcome the late detection issue of conventional methods. In fact, the development of solutions to facilitate autonomous testing at home, or assisted testing at home with the collaboration of a trained tester not requiring the qualifications of a health professional, may significantly enhance the quality and quantity of collected data and facilitate the implementation of or longitudinal follow-up studies in the future.

There are several theories and models to infer the degree of adoption of a particular technology. The Theory of Reasoned Action (TRA) (Yousafzai, Foxall & Pallister, 2010) indicates that the eventual use of a system or service is based on the user’s attitude towards it. In the same line as TRA, several models and theories were proposed to infer the eventual behaviour when new technological services or systems are adopted according to some premises on the attitude of end users towards the introduction of technologies in their lives. Examples include the Technology Adoption and Usage model (Bagozzi, 1990), the Motivational model (Davis, Bagozzi & Warshaw, 1989), or the Information Technologies Usage model (Moore & Benbasat, 1991), among others.

Each of the models enumerated above follow a different set of initial assumptions to predict the eventual use of a given technology. One of the most popular models, and the one adopted in the study discussed in this paper, is the Technology Acceptance model (TAM). This model is based on TRA, but it can be considered more flexible to be adapted to different population samples. TAM indicates that the attitude towards a given technological system is determined by two subjective variables, namely perceived ease of use and perceived usefulness.

The next paragraphs introduce the methodology followed in this study, which included a pilot experience involving 62 real users in a real scenario. The outcomes of this pilot are presented and discussed after the methodological introduction. To complete this discussion, some relevant limitations are also identified. Finally, the results obtained are summarized together with the identification of ongoing and future research lines.

## Materials and Methods

As pointed out above, this study was supported by a pilot project involving 62 senior adults. Written consent was collected from all participants in accordance with the provisions of Spanish regulations (Ministry of Justice, 2008). The official ethical committee confirmed to the authors of this study that no explicit approval was required. In no circumstance health or medical information was requested or captured from the participating volunteers. The only information obtained from participants refers to their experiences and perceptions when playing with the games in the pilot.

No control group was involved in this study. This decision was taken because no actual cognitive performance or cognitive improvements would be measured. Our aim was to detect if the technology proposed would generate a rejection attitude in elder adults. A control group would be necessary in case that we wanted to compare the results obtained in terms of some evolving variable (e.g., cognitive status). In our case, the control group would not use the platform, and therefore would be unable to provide information about their perceptions on ease of use or usefulness.

Being aware of the relevance of the technological acceptance of the system proposed, and due to senior adults being more prone to show an initial rejection attitude towards the introduction of new technologies, we introduced the Technological Acceptance Model (TAM) (Lee, Kozar & Larsen, 2003) to study this aspect. The TAM model was applied to elucidate the participants’ subjective perceptions about the willingness of using a TV-based platform if it were available to carry out cognitive training and assessment. According to this model, we surveyed the participants about the perceived usefulness and perceived ease of use. The perceived usefulness (PU) refers to the extent that a given individual believes that, using a particular technological system, his or her performance would improve. Simultaneously, the perceived ease of use (PEOU) provides an indication about the extent that a given individual believes that, using a particular technological system, the effort required would be reduced. In a nutshell, PU is about performance, and PEOU is about effort required. Low PEOU and high PU would mean that a given system is perceived as difficult or complicated to use, but facilitates the completion of many tasks in an efficient way; while high PEOU and low PU would mean that the system is easy to use, but also useless. Note that the two parameters above (i.e., PU and PEOU) are inherently subjective. Nevertheless, in our study we also monitored participants’ interactions by means of the activity logging facility available at the platform’s backend. This enabled us to check participants’ answers against their actual interactions.

### Participants and experiment design

**Figure 1:**
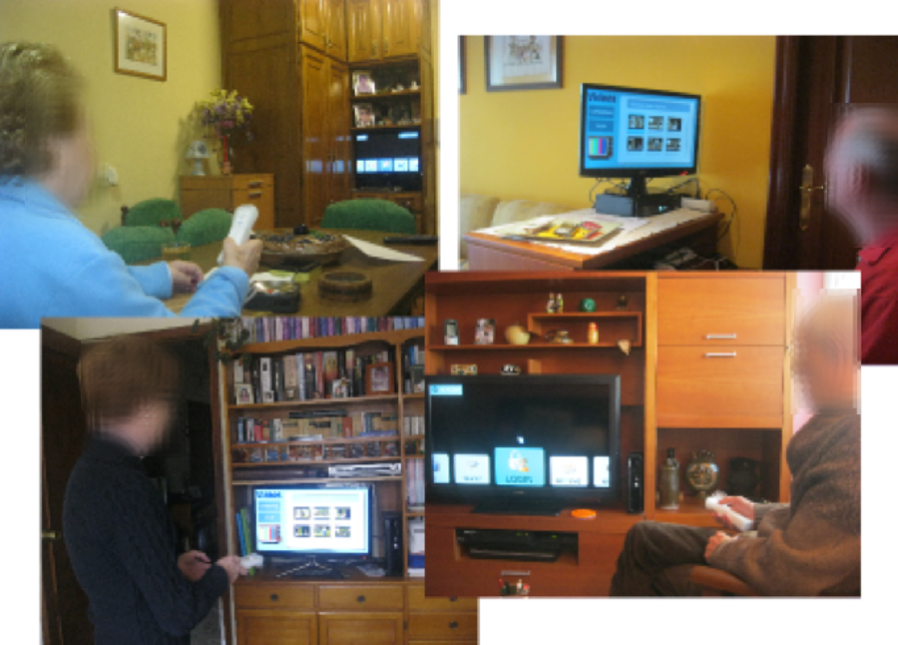
Pilot testing was performed at participants’ premises. (Photo credit: Carlos Rivas Costa).

A total of 62 subjects were selected (c.f. Fig. 1) among volunteers affiliated to the Third Age Lecture Rooms of Galicia—ATEGAL association. Although the pilot test performed in this research is exempted from an IRB approval requirement under Spanish regulations, we did receive approval for the pilot from ATEGAL. Indeed, its collaboration was guaranteed because they found no ethical, organizational, technical or any other issue that might prevent the involvement of ATEGAL users. This entity (http://www.ategal.com) is an independent legally registered association providing continuing education to senior citizens in Galicia, Spain.

Participating users had the platform at their disposal during a period ranging from seven to 15 days. Participants had to be at least 65 years old (i.e., retirement age in Spain at the time of the pilot), and have a broadband Internet connection at home. Internet connection was required because, as discussed above, participants’ interactions would be logged at a central server to compare their actual interactions with their subjective perceptions (e.g., whether the preferred game was the game actually most played or to make sure that they had actually used them).

Gender distribution was 50–50%, and participants were scattered around the region of Galicia, Spain. This region is characterized by an aging population and being a mostly rural area as compared with the rest of Spain, with near 50% of the population living in rural areas. Participants in this study were selected according to the geographical distribution of the Galician population, trying to involve a significant number of participants from both rural and urban areas. All candidates that expressed their willingness to participate were included in this study (i.e., a selection/exclusion process was not needed). Table 1 below summarizes the participants’ geographical distribution, gender and age distribution.

**Table 1:** Participants’ gender and demographic data.

Participants’ Gender	Male	Female
	(31) 50%	(31) 50%
Demographic	Urban	Rural
	(34) 54.84%	(28) 45.16%

Participants’ Age	65–70 years old	70–75 years old	75–80 years old	80–85 years old	85–90 years old	90+ years old
	(24) 39%	(16) 26%	(10) 16%	(7) 11%	(4) 6%	(1) 3%

### Materials

To guarantee common deployment conditions (i.e., common evaluation settings), the platform was implemented in a home theatre personal computer (HTPC) connected to a television set. This solution enabled us to convert any existing TV set, regardless of its age or underlying technology, into a standardized smart TV set.

To collect usage data and users’ perceptions, two questionnaires were distributed, one to be completed before the pilot and a second one to be delivered right after it. Participants filled in the questionnaires with the assistance of the staff implementing the pilot in face-to-face sessions at participants’ premises. Contact details to communicate with technical staff were provided to all participants (i.e., a phone number and electronic mail address) in case they had any doubt or trouble when accessing or interacting with the platform. No incidence was communicated during the pilot.

Besides, 36 months after the pilot test took place, a new survey was carried out to obtain some indication about the long-term effects of the experience discussed above. In this case, questionnaires were completed by phone, and were carried out by the same staff doing the initial survey.

With respect to the introduction of serious games to support neuropsychological assessment, four general approaches can be found in the literature for the *gamification* of the cognitive tasks aimed at capturing the subjects’ (i.e., players’) cognitive performance.

1. Take an existing game and use it as a platform for creating cognitive measurable tasks by modifying game parameters. For example, the classic redemption game Whack-a-Mole (Delaney, 2009) captures different measures such as the speed and the deviance from target. This approach takes this well-known existing game and ‘hooks into’ its mechanics to capture players’ performance. The execution of this approach requires a good recognition of the particular cognitive abilities that are tapped by concrete tasks in video games.
2. Mimic the testing mechanics of a paper-based test trying to be challenging and fun at the same time. Differently to the first approach above, in this case the starting point is a traditional neuropsychological assessment suite, and the objective is to create a video game that has the same validity by replicating its mechanics.
3. Embed already computerized neuropsychological tests into a virtual reality environment.
4. Replicate real life situations using virtual reality environments that try to depict realistic situations like car driving in a city (Plancher et al., 2012), an apartment (Kurtz et al., 2007), or a supermarket (Zygouris et al., 2015; Klinger et al., 2004; Tam et al., 2005) among others.

Other approaches might be possible, such as to design a videogame from scratch embedding cognitive tasks aimed at capturing performance data to enable an eventual assessment of a selection of cognitive areas. However, no practical examples were found at the time of writing this paper.

In our case, the games introduced can be classified into group 1 above. The selection, design and implementation of the game collection were carried out in collaboration with ATEGAL. An occupational therapist appointed by ATEGAL provided advice to the research group in the identification and selection of a set of cognitive-related activities, which were eventually implemented as games for our platform. Among these games, four of them were specifically targeted to neuropsychological stimulation, which will be further discussed below.

**Figure 2:**
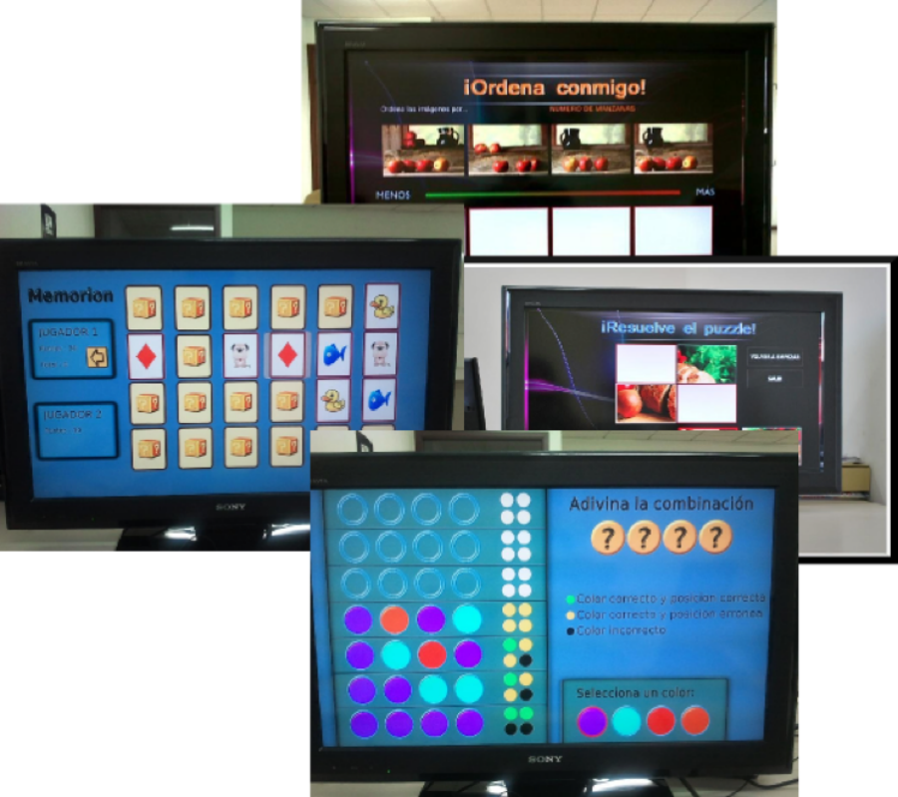
The games used in this study are adaptations of classical games (Photo credit: Carlos Rivas Costa).

The possibility to assign scores to user interactions was also taken into account when selecting and implementing the games mentioned. Besides their usefulness to potentially perform cognitive evaluation, these scores would also be visible to other users participating in the pilot. The games implemented were (cf. Fig. 2):

• *Memorion.* Each user has available a limited number of pairs of cards (i.e., every card has a duplicate card). At the start of the game, all cards are presented facedown, and users have to flip them one by one to discover all pairs of cards. In turn, each user selects two cards in sequence. If both cards are identical, one point is scored and the selection of cards is repeated again. In case the cards selected are different, cards are flipped again and the turn is passed to the next player. This game is intended to assess short memory capabilities.
• *Find the Intruder.* In this game, participating subjects are presented with a collection of images and they have to identify which one does not belong to the collection. During the game, images are randomized to prevent the apparition of presentation patterns and thus users from recognizing them. This game addresses the perception, decision-making, association and categorization capabilities.
• *Sequences.* Users are presented with real situations where a sequential relation occurs. This relation may be numerical, temporal, cause–effect, etc. The correct sequence of events in each situation is modified randomly and presented to the player, who has to place the events again in the correct sequence. The presentation of both individual events and sequences is randomized to prevent presentation patterns.
• *Puzzle.* Users shall complete a series of graphical puzzles. An image is divided into puzzle pieces and those pieces are shuffled. As users solve puzzles, their difficulty (i.e., number of pieces) is increased. Users are penalized in case they made a wrong selection.
• *Questions and Answers.* Users are challenged with questions about an image surrounded by additional images. Users shall provide the correct answer to the question by selecting one of the images provided.

With respect to the access and interaction with the games proposed, the process was fully self-administered. Users did not receive any help from third parties to interact with the games. However, they did receive initial assistance to access the system and navigate it to reach the games to interact with them.

### Data collection and processing

The pilot test was organized into two phases involving 42 and 20 subjects respectively. Seven copies of the platform were available to implement the pilot. Therefore, to facilitate pilot logistics, clusters of at most seven users were defined according to their home locations. Then, one copy of the platform was installed in each of the homes in one cluster, and when the pilot was completed there the whole setting was transferred to the next cluster.

Volunteers did not receive training until the date when the system was installed in their premises. During the first visit, a member of our technical staff explained to the participating user the layout of the user interface and how to interact with it. As indicated above, participants were provided with the contact details of our technical staff to solve any situation that may appear during the pilot. From this first visit until the end of the pilot, participants would interact with the system in a totally autonomous way.

Once participating users were logged into the system, any interaction performed by them would be transmitted in real time to a central server. Interaction data would be eventually used as a reference once users’ perceptions were captured using questionnaires at the end of the pilot. Communications between users’ premises and the central server were encrypted.

As pointed out above, interaction data was used for for the validation of user perceptions. These perceptions and other subjective information collected using questionnaires was checked against the information generated and automatically sent to the central server, and also against the information generated from the interactions of other participating users. In other words, the aim of this strategy was to compare opinions in relation to the perceived ease of use and usefulness with actual interaction data, and therefore to have objective evidence to validate the subjective information obtained from users. In fact, cross-checking enabled us to confirm that the information provided by volunteers in their responses to the questionnaires matches the automatically captured interaction data.

## Results

In this section we discuss the main results obtained after completion of the pilot study. According to the objectives of the TAM model, the results obtained on perceived usefulness by users and ease of use are discussed below. Finally, the results on the favourite devices to interact with serious games are also presented.

**Figure 3:**
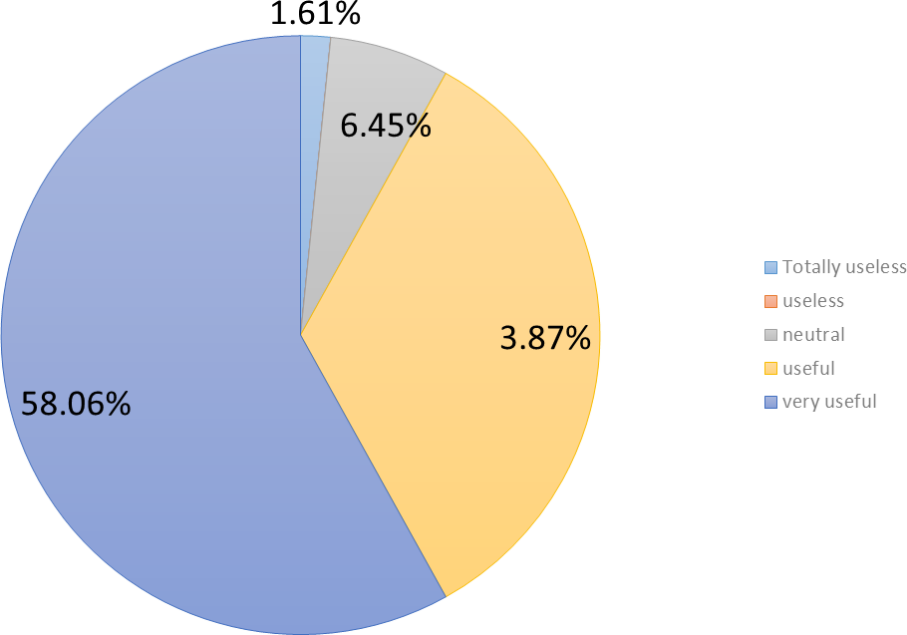
Perceived usefulness.

Participants were asked about the perceived usefulness of the games implemented. A 58.1% (*n* = 36) of them perceived those games as very useful and a 33.9% (*n* = 21) of them as useful (cf. Fig. 3). In other words, more than 90% (*n* = 57) of participants declared that the games were not just an entertainment option, but also a means to check their memories and their reasoning capabilities.

**Figure 4:**
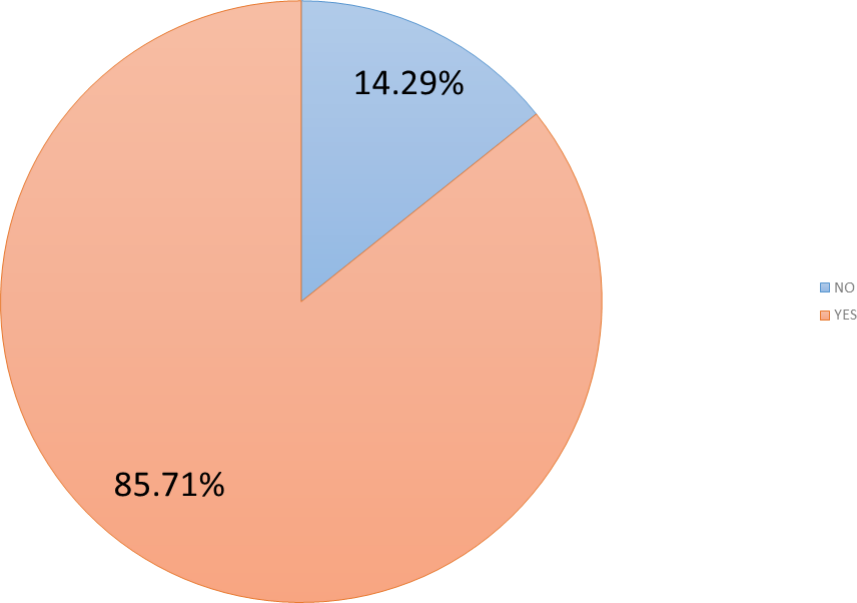
Long-term perceived usefulness of cognitive games.

Besides 36 months after pilot testing a new survey was performed among the participants in the original experience. Due to different reasons, only 21 individuals participated in the survey from the original group of 62 participants. In this case, participants were asked again whether they perceived that the use of serious games at home could have been useful. (cf. Fig. 4). 86% of participants (*n* = 18) had this long-term perception. This answer is confirmed when they were asked whether their cognitive state could improve if they continued using the platform after the pilot, with a 95.2% (*n* = 20) of positive answers.

**Figure 5:**
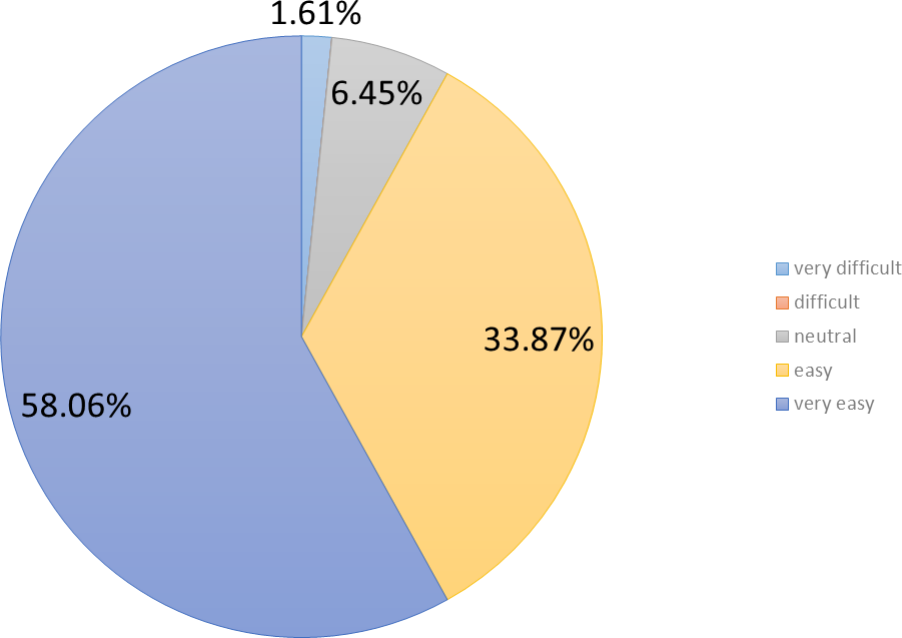
Perceived user-friendliness of the TV-Based game platform.

Secondly, they were also inquired about the perceived ease of use of the TV-based game platform. In this case, the 92% (*n* = 57) of users declared that it was very easy or easy to play with the aforementioned games (c.f. Fig. 5).

**Figure 6:**
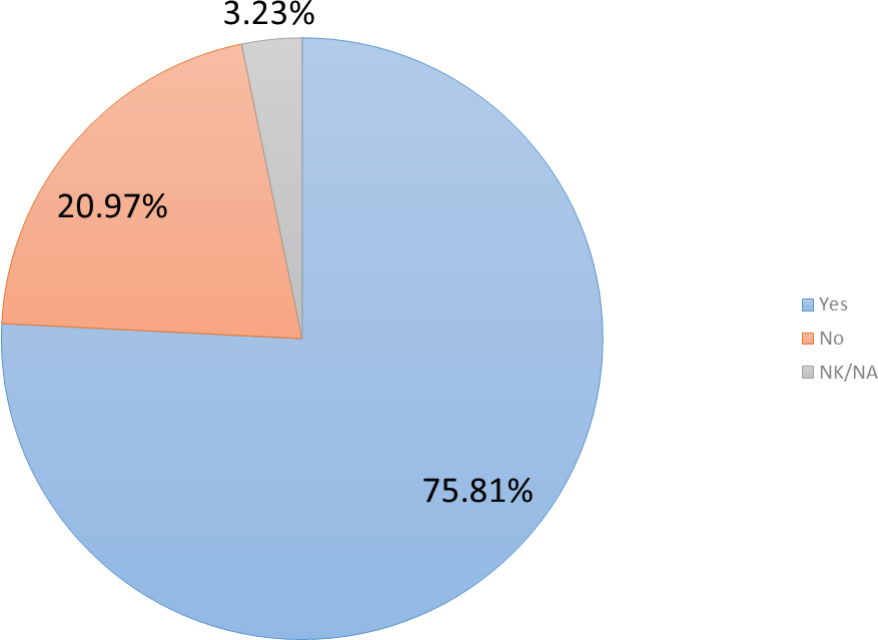
Participants’ willingness to keep using the system after the pilot study.

Participating users were also questioned about their willingness to keep using the platform after completing the pilot study. Most of them (i.e., 76%—47 users, cf. Fig. 6) would keep using the system installed at their homes.

**Figure 7:**
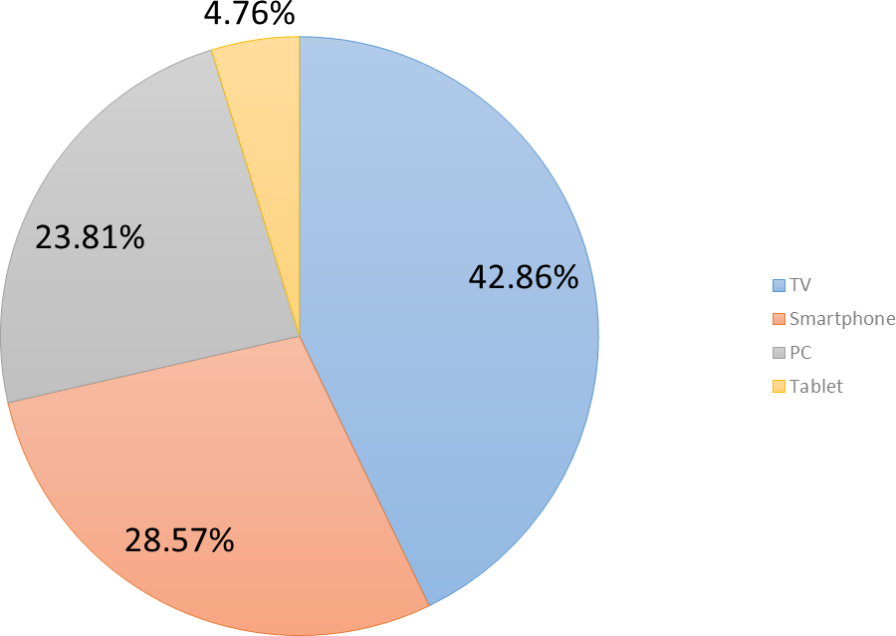
Preferred device to interact with the cognitive games in the pilot.

Finally, participants were questioned on their preferred devices to interact with the games available during the pilot, and more specifically on which devices they perceived as more accessible and appropriate to interact with them. Four options were proposed; namely, a TV set, a smartphone, a tablet computer or a PC (cf., Fig. 7). In this case, the preferred user device would be the TV set (42.9% (*n* = 9) of participants), followed by a tactile device—smartphone or tablet (33.3% (*n* = 7) of users)—and finally the traditional PC (23.8% (*n* = 5)). This is confirmed by the fact that a TVset is perceived as more accessible than a PC (76.2% vs. 23.8%), while the PC is the device considered as less appropriate to interact with in these kind of games.

**Figure 8:**
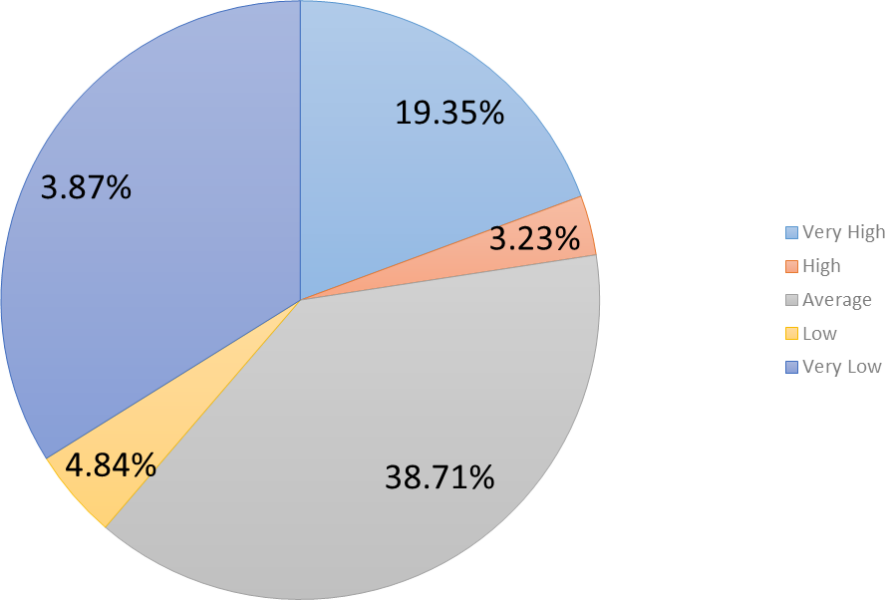
Willingness to use a PC to interact with the cognitive games in the pilot.

Participants were also asked about their willingness to interact with the games in the pilot in case they needed to use a standard PC (cf. Fig. 8). 38.70% (*n* = 24) of participants declared that it was very low or low; 38.70% (*n* = 24) expressed a neutral position, and 22.6% (*n* = 14) of respondents declared that it was very high or high.

## Discussion

This study confirmed that senior adults show a high acceptability degree of the proposed TV-based game platform. Perceived ease of use is over 90%, which supports the conclusion that the digital divide typical in our target population group will not justify the rejection of the introduction of serious games for cognitive evaluation when this solution is introduced using a familiar device such as the ubiquitous TV set. Note that only 24% would interact with serious games using a personal computer, even when this PC would be placed at home or outside a clinical facility. In other words, our initial hypothesis on the use of the TV set as an effective device to carry out cognitive evaluation using serious games is confirmed by the evidence obtained during the pilot experiment discussed in this paper.

The literature reviewed in relation to the introduction of serious games for cognitive evaluation discusses approaches mostly based on the use of personal computers. The final aim of these studies is to demonstrate that serious games can indeed be used for cognitive evaluation. Technological acceptability of the new proposed method is not at the core of their objectives and, therefore, not discussed in depth. Most works analysed declare that tasks included were adapted to final users, taking into account primarily their cognitive capabilities, but providing no special attention to technological issues. For example, task difficulty is typically adapted by adjusting the number of stimuli or their presentation speed (Delgado et al., 2016; Tong & Chignell, 2014), or the type of navigation that the user can perform (Salgado-Pineda et al., 2016; Armstrong et al., 2013; Plancher et al., 2012). With respect to the interaction device utilized, the TV set is only marginally present when compared to other devices like the personal computer or the mobile phone. However, the TV set is acknowledged as a device closer to senior adults and their daily activities (Sauzéon et al., 2016a; Sauzéon et al., 2016b). Other studies propose a tactile interface as the most adequate interaction mechanism for elder people (Fukui et al., 2015; Deguchi et al., 2013) due to their ease of use, which does not require fine-grained motricity, even for patients with cognitive limitations and motor dysfunction. However, in the field of ICT solutions based on games for cognitive evaluation, no published results are available at the time of writing this paper discussing the evaluation of the user experience, neither based on TAM nor in other applicable methodologies. Finally, and considering that most solutions available at this time are designed taking the personal computer as the reference interaction device, we found in our study that 39–34% very low + 5% low of the senior adults participating would not participate in a serious game experience when a PC is used to interact with them. This is a relevant result for those considering to further develop this promising instrument for cognitive assessment.

Meanwhile, the introduced TV-based game platform do not cause usability or rejection attitudes related to the existing technological gap among the senior adult population. On the contrary, thanks to the introduction of ICT, a solution like the one discussed in this paper facilitates adaptability and personalization to the needs of specific users. Thus, the fact that senior adults are very heterogeneous insofar their physical and cognitive limitations are concerned has to be considered. Consequently, TV appliances or mobile phones have certain mechanisms of adaptation to the needs of the elderly (e.g., adaptation of access controls, interface configuration, text format and layout, or sound levels).

We have validated our hypothesis on the acceptability of the TV set to support the interaction with serious games. A total of 91.23% of participants declared that the TV set is perceived as a device with which is very easy or easy to interact. This opinion is held over time, after using the serious games in the pilot and even as a long-term perception. In fact, 36 months after the pilot, when participants were asked about which device would be preferred to interact with serious games for cognitive assessment, the TV set, phone, tablet and PC being the options proposed, 43% declared that they wanted to used them on the TV, even when 66% of the participants had experience using a PC. The TV set is the preferred device for interacting with serious games.

It is also worth noting that 28% would use them on a smartphone as a first option. This is a relevant result that in our opinion is influenced by the fact that many users already participated in training workshops in the premises of ATEGAL, the institution supporting this pilot and providing users. In any case, it shows a clear trend towards the digitalization of senior adults and the reduction of the digital divide among our elders. Researchers need to carefully look at tactile devices, such as smartphones and tablets, as there is an increasing percentage of the elderly population that already feels comfortable using these devices.

Our second research question was also answered. During the pilot we also obtained evidence in the sense that such a setting (i.e., TV device + serious games) may serve as a mechanism that would eventually support an ecological screening instrument for mild cognitive impairments, as participants perceived it as useful both during the pilot and several months after completing it, and declared that they (i.e., 76% of participants) would continue using it. Also, according to Quinn et al. (2015), the apparition of memory-related impairments is among the most relevant concerns for senior adults and their caretakers. In this regard, this study also evidences that senior adults can become an active part of their own care by means of solutions like the one discussed in this paper, as they are perceived as usable and acceptable for this population group. Thus, evidence obtained shows that the system introduced facilitates the realization of longitudinal, non-invasive studies for the early detection of cognitive impairments since there is no rejection among the target population to keep this assessment instruments at home. In this regard, the discussed TV-based game platform may also contribute to avoid the late diagnosis drawbacks associated to classical neuropsychological testing. To sum up, the development of solutions to facilitate autonomous testing at home, or assisted testing at home with the collaboration of a trained tester not requiring the qualifications of a health professional, would significantly enhance the quality and quantity of collected data and facilitate the implementation of longitudinal studies, both at individual and collective level.

The practical implications of using serious games as an accepted instrument for cognitive assessment are very relevant. The early detection of Mild Cognitive Impairment, for example, leads to a better treatment and the reduction of the pace at which the deterioration evolves (Leifer, 2003). This type of instrument can be easily self-administered, which leads to huge savings in clinical costs. Cognitive assessment at home is relevant not only from a medical point of view, but also from a social perspective. The quality of life, which in turn may affect the neuropsychological status, depends upon many factors beyond health conditions. The World Health Organization defines wellness as “a state of complete physical, mental and social well-being, and not merely the absence of disease and infirmity” (Grad, 2002). Socialization is an essential requirement, in particular for elders that live alone (Perissinotto, Cenzer & Covinsky, 2012). Thus, as a consequence of the technical acceptability analysis carried out (i.e., usefulness, ease of use and accessibility perceived by end users), we can confirm that there is at least initial evidence about the convenience of using the TV set to introduce games played as a means to assess the cognitive status of senior adults at home. Besides, the TV set offers a much more familiar interface for many users (Vázquez et al., 2012; Ofli et al., 2016; Heart & Kalderon, 2013) overcoming the digital divide when using an ICT-based health and care systems at home.

## Limitations

No matter the highly positive acceptability of the approach discussed in this paper, several limitations were detected that require further analysis.

Firstly, the design and deployment of a self-administered, home-based cognitive-testing facility targeted to senior adults involves important challenges. Among the most relevant is to avoid the introduction of new elements that may pose new challenges to target users (e.g., technological challenges, comprehension challenges, personalized stimuli presentation, etc.) that may affect the final outcome of self-administered testing. In this regard, further research is needed to guarantee the reliability and validity of self-administered cognitive testing.

Secondly, a clinically sound study would require a wider sample with a representative distribution attending to relevant parameters such as age, gender, previous education, urban/rural character, etc. Nevertheless, a pilot experiment involving 62 senior adults already has a relevant statistical significance (Grissom & Kim, 2006) to assess the acceptability of a TV-based game platform for cognitive assessment.

Finally, during the new survey carried out 36 months after the original study, participants were explicitly questioned whether they perceived that the original experience had improved their cognitive state. Although 86% of participants declared that they indeed had that perception, the lack of a control group prevented us to corroborate this subjective perception of the volunteers’ cognitive improvement. Besides, note that this answer might be biased because original users with a deteriorated cognitive state at the time of carrying out the new survey would not be among the respondents. That is, deteriorated cognitive state is one of the causes of the limited number of participants in the second survey (21 vs. 62). Obviously, authors do not claim that TV-based cognitive stimulation or training at home provides a cure to cognitive impairments, but only that there is initial evidence about the technical acceptance (i.e., usefulness and ease of use) of home platforms, and more specifically of games, as a means of cognitive evaluation.

## Conclusion

In developed countries, senior citizens represent a growing part of the population, and due to an ageing population the incidence of cognitive-related impairments is higher and higher. However, today, there is still no adequate approach tackling the early detection of such medical conditions.

In this research we studied the possibility of introducing games to help users to assess their cognitive status by means of a solution deployed around the ubiquitous TV set. Participants in this experience provided evidence on how older people who have interacted with a TV-based system confirmed their initial perception about the ease of use of typical Internet services. For them, the TV set is much simpler, much friendly, and causes a much lower rejection attitude than other technologies such as computers, tablets or smartphones. The validation of the hypothesis of the technical acceptance of digital services on the TV set is another relevant contribution of this work.

This research does not try to rigorously validate the proposed approach for the actual cognitive evaluation in the elderly, according to clinical standards, but to assess the acceptance of such a technical setting for that purpose by elder adults. Note that, in a hypothetical scenario where this claim were not confirmed, it would be difficult to justify further investigations on how to assess the cognitive status of elder adults at home with such technical setting. The claim being confirmed means that it makes sense to introduce such technical settings in users’ premises with the aim of performing their cognitive evaluation, assuming that indeed more research is required to validate such a tool.

To sum up, the study did confirm the technical acceptance (i.e., usefulness, ease of use and accessibility perceived by end users) of games in a TV-based platform as a means of cognitive evaluation. Nevertheless, in spite of this promising initial evidence, more research is needed in order to implement serious games in a way that they are widely accepted by the medical community as a valid, reliable way to perform cognitive evaluation at home.

Indeed, and taking into account the high end-user acceptability figures achieved, future work shall be focused on the development of a series of digital games to support cognitive assessment built on sound psychometric and clinical foundations. Note that the studies referenced on cognitive evaluation games are still in an early development stage and require some advances from a neuropsychological point of view to guarantee their predictive and discrimination characteristics for the cognitive areas addressed (e.g., episodic memory, executive functions, attention, language, etc.). More specifically, games shall comply to the quality indicators established by APA (Eignor et al., 2013) both at the reliability level (i.e., test-retest, parallel forms, internal consistency and inter-rater reliability); validity level (i.e., criterion, content, construct, face, external and ecological validity); normative level (i.e., validation shall be performed using a wider and more representative population sample to support an statistically significant extrapolation to the western European population), and finally at the analytical level (i.e., data extracted from games shall be deeply analysed taking advantage of the potential of novel artificial intelligence and machine learning techniques, such as classification trees or CART or Random Forest regression, rather than just considering classical correlation and/or linear regression).

##  Supplemental Information

10.7717/peerj.2845/supp-1 Data S1 Raw data from perception surveys (first round)

10.7717/peerj.2845/supp-2 Data S2 Raw data from perception surveys (second round)

## Additional Information and Declarations

### Data Availability

The following information was supplied regarding data availability: The raw data has been supplied as a Supplemental File.
